# Improving Experimental Designs for Interventions to Reduce Intimate Partner Violence: Protocol for Refinements to Single-Case Experimental Design for a Safety Planning Intervention in Ontario, Canada

**DOI:** 10.2196/67450

**Published:** 2025-12-12

**Authors:** Patricia O'Campo, Nicholas Metheny, Pearl Buhariwala, Shajitha Rasiah, Alexa R Yakubovich

**Affiliations:** 1 MAP Centre for Urban Health Solutions, Li Ka Shing Knowledge Institute, St Michael's Hospital, Toronto, ON, Canada; 2 Dalla Lana School of Public Health, University of Toronto, Toronto, ON, Canada; 3 Nell Hodgson Woodruff School of Nursing, Emory University, 1520 Clifton Rd. NE, Atlanta, GA, 30322, United States, 1 5402472331; 4 Department of Community Health and Epidemiology, Faculty of Medicine, Dalhousie University, Halifax, NS, Canada

**Keywords:** single-case experimental design, intimate partner violence, safety planning, mHealth, safety, experimental design, partner violence, violence, protocol, planning intervention, Ontario, Canada, North America, web based, effectiveness, mental health, depression, anxiety, domestic violence

## Abstract

**Background:**

Intimate partner violence (IPV) affects 2 in 5 women in Canada, leading to both physical and mental health consequences. Safety planning is a secondary prevention intervention designed to assist those experiencing IPV in taking steps to increase their safety and decrease contact with their abusive partner. Safety planning has been shown to help survivors mitigate the negative mental health effects of IPV and encourage actions to increase safety, but evaluation outside the United States remains limited.

**Objective:**

Our team plans to evaluate the use of single-case experimental design (SCED) to assess the effectiveness of a web-based safety planning app (*WITHWomen Pathways*) for women experiencing IPV in the Greater Toronto Area. The study also explores whether women can be safely engaged for intense follow-up.

**Methods:**

SCED evaluation will involve multiple baseline and postintervention assessments of a small number of women experiencing IPV (n=6). Participants will be recruited from IPV service organizations across the Greater Toronto Area. SCED will allow for rigorous within-subject comparison, using repeated measurements at multiple time points for 3 primary outcomes (decisional conflict, empowerment to take safety actions, and use of safety strategies) and 2 secondary outcomes (mental health and IPV recurrence). The evaluation will include 5 phases: recruitment, eligibility screening, multiple baseline interviews, intervention (web app delivery), and multiple postintervention assessments. In this paper, we also discuss the development of rigorous protocols for maintaining safety and flexible data collection methods (in person, via telephone, or online).

**Results:**

Recruitment began on July 3, 2024. As of December 2025, a total of 4 participants have been recruited and have completed multiple baseline assessments. Data analysis has been completed for 4 participants, and results are expected to be published in spring 2026.

**Conclusions:**

The SCED approach may offer a novel and ethical evaluation method for IPV interventions. If effective, the WITHWomen Pathways web app could significantly improve help-seeking and safety planning among women experiencing IPV in the GTA. This study may serve as a model for future IPV intervention evaluations, demonstrating robust evaluation data and participant safety.

## Introduction

Two in five women in Canada have experienced some form of intimate partner violence (IPV) in their lifetime [1]. Such experiences include aggressive, coercive, controlling, or abusive behaviors resulting in physical harm (eg, inflammatory conditions, self-harm, HIV, miscarriage, traumatic brain injuries) and nonphysical harm (eg, anxiety, posttraumatic stress disorder) [2-4]. While IPV occurs across populations and outside male-female relationships, the most widespread form of IPV is violence perpetrated against cisgender women by current or former cisgender male intimate partners [5,6].

 Although some women can extricate themselves from violent relationships on their own, most require assistance and support to either leave the relationship or work with the partner to end the violent behavior. Liang et al’s [7] theoretical framework of IPV help-seeking addresses 2 main forms: formal and informal. Formal help-seeking includes assistance from legal, medical, religious, or social services, while informal help-seeking refers to assistance sought from family, friends, other survivors, and trusted confidantes [7]. Both formal and informal help-seeking are shown to improve the mental health of those experiencing IPV, often by enhancing the survivor’s own coping abilities and social support [7].

 Safety planning is a secondary prevention intervention designed to empower survivors to begin the help-seeking process by taking independent actions to maximize their own safety. Similar to household safety plans for natural disasters or house fires, an IPV safety plan takes into consideration an individual’s available resources, living arrangements, and needs before recommending individualized, concrete actions that can prevent a violent event or mitigate its damage. To assist women experiencing IPV to begin the formal help-seeking process, this team created *WITHWomen Pathways,* a web-based safety planning app localized to the Greater Toronto Area (GTA; the metropolitan area encompassing Toronto, Ontario, Canada and the regional centers of Durham, Halton Peel, and York). This hyperlocal app is adapted from *MyPlan,* an existing safety planning mobile app in the United States [8] and Canada [9] that has shown effectiveness in reducing decisional conflict and promoting safety planning behaviors for women experiencing IPV in traditional randomized controlled trials (RCTs). Additional details on the creation of WITHWomen Pathways and its features are published elsewhere [10,11].

Safety planning interventions are commonly evaluated via their ability to jumpstart survivors’ help-seeking processes by (1) reducing decisional conflict (a state of uncertainty when faced with choices that represent loss or conflict with personal or cultural values), (2) empowering people who experience IPV to take actions to improve their safety, and (3) providing concrete, self-directed actions that align with their goals and needs [12-14]. While there is evidence from the United States to suggest that safety planning can help improve decisional conflict and mitigate future psychological and physical violence, there have been few evaluations of safety planning interventions outside the United States [9,14-16] and only 1 in Canada [17]. This review found no differences in depressive symptomatology or posttraumatic stress disorder symptoms between users of a static, generic safety planning app and one that is more tailored to their specific circumstances. However, the tailored app (which is more similar to the WITHWomen Pathways approach) did show meaningful effects on these mental health outcomes for subgroups of women—especially those who had children in the home, those who were experiencing more severe physical or sexual abuse, or those who lived in a large or medium-sized city [9]. The latter finding may be a function of the additional resources available to women in more urban areas, which a tailored app may be more helpful at recommending.

While evaluation of safety planning interventions is key to improving effectiveness and widespread implementation, RCTs in this space are exceedingly complex due to the important safety considerations and privacy concerns regarding women who are currently experiencing IPV [16-18]. Moreover, RCTs are expensive, complex studies that require large numbers of participants, and people experiencing IPV constitute a hard-to-reach population [19]. This places them out of reach for many community-based organizations that wish to evaluate innovative, survivor-centered programming but lack the scientific expertise and resources to conduct fully powered clinical trials. Moreover, many RCTs of safety planning interventions show null effects between intervention and control groups, which is hypothesized to be due (in part) to an intervention effect in the control group—that is, asking women in the control group about their IPV constitutes an intervention in and of itself, potentially blunting the true effect of the safety planning intervention [20,21].

**Figure 1.**
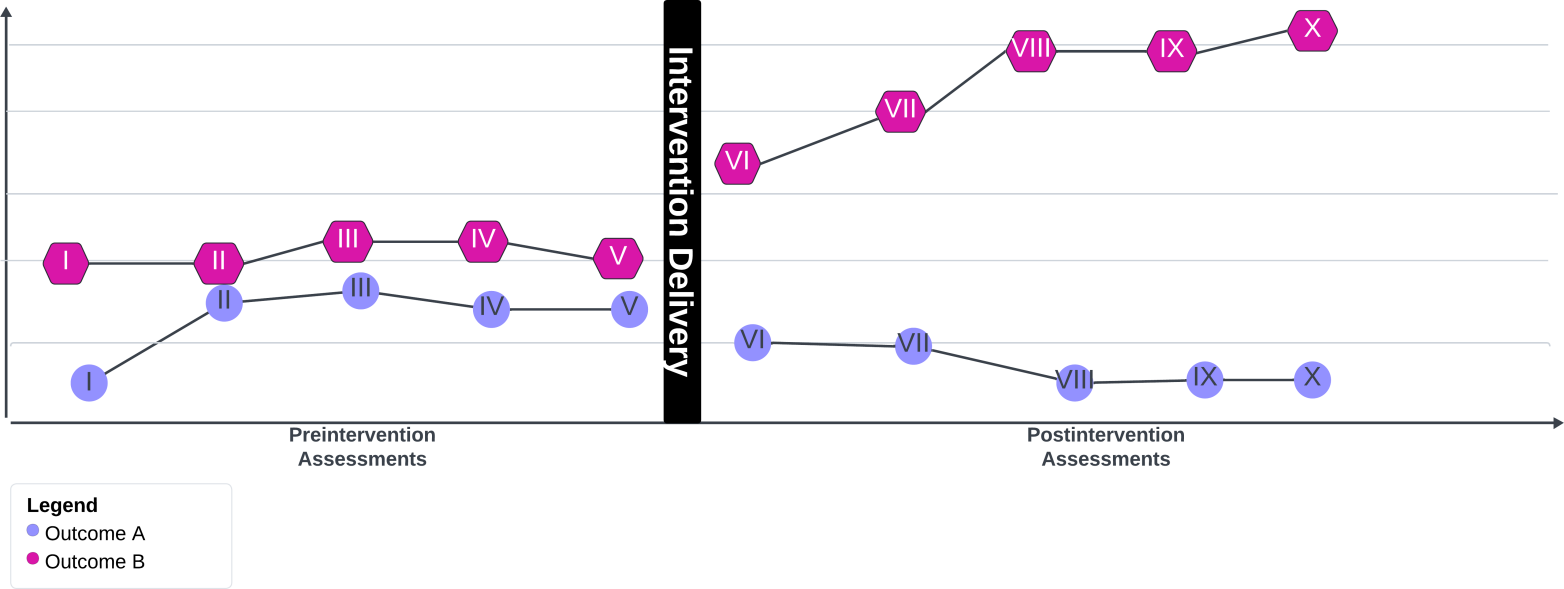
Overview of a typical single-case experimental design study flow.

Single-case experimental design (SCED) is a methodology that allows us to build on findings in a cost-effective and ethical way without unnecessary duplication via another large-scale, expensive RCT. As funding for research continues to decrease in many countries, such RCTs can be a drain on finite resources and impede scholarly progress [22,23]. To overcome some of the challenges posed by traditional evaluation methods, we are interested in using a potentially more rapid and efficient evaluation method that adapts the SCED clinical methodology, alongside novel safety protocols, to the evaluation of WITHWomen Pathways. SCED uses multiple observations in the same participant at short intervals (the time between observations depends on the outcome of interest) both before and after the introduction of the intervention (see Figure 1) [9,24]. Statistically nonsignificant differences in the outcome measures of interest between preintervention visits indicate stability and establish a preintervention baseline that is then compared to the subsequent postintervention time points [25-29]. After the intervention—which may be a single session or consist of multiple sessions [30,31]—is delivered, data collection over multiple postintervention time points allows for the establishment of behavioral trajectories that illustrate when and how change happens. In other words, the participant’s baseline measures act as the control. When multiple SCED cases are aggregated, SCED can explain small but meaningful causal variations in intervention effects using fewer participants than an RCT [28].

SCED has been used in medical, psychological, and behavioral research for decades to better understand the mechanisms of intervention effects. Recently, it has also been used to assess the effectiveness of technology-based interventions in other areas [32], for example, in vulnerable populations such as adolescents experiencing psychological distress [33], individuals with serious mental illness [34,35], adults with severe autism spectrum disorder [36], and young children [37]. The extant literature suggests that SCED is a viable alternative to RCTs, especially for sensitive outcomes, because it allows for closer observation of individuals [38]—something which is critical for ensuring the immediate safety of participants living with IPV [26,27]. However, to the best of our knowledge, this will be the first adaptation of the methodology to IPV studies [32].

To gauge whether SCED is a viable approach for evaluating a web-based safety planning tool for English-speaking women in the GTA, we have established the following study objectives:

1. To understand whether women who are living with or have recently experienced IPV can be safely engaged for intense follow-up for the purpose of evaluating a safety planning intervention
  - We hypothesize that our safety protocols and individualized approach will allow for safe participation in the study, as measured by a lack of reportable or adverse events.
2. To examine the preliminary effectiveness of WITHWomen Pathways and our novel safety protocols on 3 primary and 2 secondary outcomes of interest
  - We hypothesize that within-person changes to decisional conflict and depressive symptoms will show a decrease, whereas empowerment to take safety actions will demonstrate an increase.

## Methods

To our knowledge, SCED has never before been applied to IPV evaluation; therefore, the methods in this study involved developing novel safety protocols for women engaging with high-touch research while at high risk for IPV, as well as data collection and analysis procedures.

### Ethical Considerations

This study and all relevant protocols were reviewed and approved by the St Michael’s Hospital Research Ethics Board (REB; #20‐120). All study procedures comply with the ethical standards of the St Michael’s Hospital REB and follow the principles outlined in the Declaration of Helsinki and relevant Committee on Publication Ethics (COPE) and JMIR guidelines. Further details on informed consent, privacy, and confidentiality are provided in the following sections. Below, we review the development of safety protocols before describing the evaluation of the WITHWomen Pathways app.

### Universal Safety Considerations

In-person interviews will be conducted in complete privacy, except for participants who are accompanied by children younger than 2 years. If privacy cannot be ensured, interviewers will reschedule the interview for a different time or place, allowing participants the flexibility to choose a safer or more convenient option. For telephone interviews, interviewers will confirm before beginning the interview that participants can talk privately and safely. Interviewers will be trained to detect whether someone else is present or listening in on the call, using cues such as speech hesitations or background noises, and offer to call back if needed. If a participant is not alone, the interviewer will switch to asking nonsensitive, health-related questions as a safety measure. The online surveys will contain a preamble with online browsing safety tips, including specific recommendations to ensure the participant is alone before beginning the survey and instructions on how to use a private or incognito browser and how to quickly exit the survey. Furthermore, the WITHWomen Pathways app itself has safety features that participants can use should their abusive partner be made aware of their use of the app. As a mobile-friendly web app, WITHWomen Pathways is not physically downloaded, making it easier and quicker to remove any traces of its use. It also contains a quick exit button, which links to an innocuous website (currently hgtv.ca). In addition to these in-person and asynchronous safety measures, all participants completing the screenings by phone will be asked to confirm whether they feel that they are in a safe space, before moving forward. As rollout continues, the safety of completing interviews online will be considered and adjusted as necessary.

### Safely Contacting Participants

Safety protocols are of paramount importance in this study and necessitate bespoke approaches to safety strategies. To develop these strategies, we first consulted the literature and drafted a tentative protocol. These strategies include maintaining a general conversation during phone calls before discussing the research study until it is confirmed that the participant is in a safe environment, conducting regular check-ins to ensure the participant’s comfort and well-being, and sending an email prior to making phone calls to confirm that the scheduled time is still safe for the participant.

Our team’s peer advisory committee (comprising survivors and IPV advocates) provided feedback on the draft protocol and recommended changes to recruitment and contact procedures (eg, specifically mentioning IPV on recruitment flyers). During the consent process, participants will also be provided with an optional safe contact form where they can list up to 3 “safe contacts” who will only be contacted if the researcher cannot reach the participant. The purpose of this form will be communicated to participants as a standard procedure to ensure their safety if they cannot be reached. Safe contacts will be informed that the participant is part of a women’s health study and has given permission for the researcher to contact them if needed. Participants will be encouraged to inform their safe contacts about the possibility of being contacted by St Michael’s Hospital in the future. The participants will be asked to sign the safe contact form at baseline to ensure a thorough understanding and to provide written consent for contacting the listed individuals. Information requested for each safe contact includes name, relationship, phone number, and email. Participants will be asked to prioritize whom to contact first and to specify the preferred method of communication. The safe contact form will be reviewed and updated with participants at baseline and during follow-up to maintain accurate information.

### Follow-Up Communication Model

A communication protocol will be established between researchers and each participant, specifying when to expect contact from staff, ranking the preferred modes of safe contact (eg, email, phone, and mail), and choosing a code word to end communication if the environment is not private or safe. This information will be reviewed with participants at the first baseline and first follow-up assessments in case their situation changes. At each contact point, participants will be assured that their safety and confidentiality are prioritized and that the research team cares about them as individuals.

### Recruitment

While previous app-based safety planning interventions have attempted to reach women experiencing IPV who are unready to be connected with formal services [24], our community partners are interested in using WITHWomen Pathways as a complementary resource for those who present to formal services but are either unready (eg, ambivalent) or unable (eg, due to the presence of pets, male children, or substance use) to avail of temporary housing services or those who are unsure whether they need formal services. Therefore, participants will be recruited through a network of nearly 2 dozen organizations that provide violence against women services across the GTA. Prior to recruitment initiation, the research coordinator (SR) sent emails to staff at each organization about the purpose of this research and ensured that we were in agreement and alignment on participant safety and protocols for safety management, as well as the inclusion criteria. The organizational leadership has agreed to take part in the SCED evaluation study, allowing us to move forward with the recruitment process. To recruit participants, the research coordinator will share flyers and information sheets with the leadership, who will then share the materials with clients and others in their network via their own email listservs and social media accounts.

### Eligibility Screening and Informed Consent

Once a woman engages with recruitment material, the research coordinator will reach out using the participant’s preferred mode of contact (ie, email or phone) to invite her to learn more about the study and complete the eligibility screener. At the start of this discussion, participants will be provided with a “quick exit” strategy whereby the topic of conversation can quickly move to a safe subject in case privacy is breached. For example, study staff will inform potential participants that if either she or the interviewer notices that privacy is breached, they can quickly switch to talking about a “safe” topic of choice as determined by the participant (eg, shopping, cooking, children, sports, or movies).

The screener consists of questions about basic sociodemographic information (eg, age, residence, and sex), and the level of current or recent IPV is determined via the 9-item WITHWomen screener [39]. The target population for the SCED evaluation of the WITHWomen Pathways safety planning app is 6 self-identified women in the GTA who are currently experiencing physical, sexual, or emotional IPV or controlling behaviors or had these experiences in the past 12 months. Participants will be eligible if they are able to read, write, and speak English; identify as a woman; are 18 years or older; and reside in the GTA. Women who have custody of children younger than 13 years will also be eligible; this age was chosen to reduce the likelihood of multiple abusive partners (eg, presence of dating violence among adolescents) and better understand how women with young children make safety decisions. Men, individuals living outside the GTA, those with children older than 13 years, and those who are experiencing no IPV will be ineligible. Women who do not meet the eligibility criteria but who are experiencing IPV will be immediately referred to formal sources of support (eg, a domestic violence shelter, a hotline, or law enforcement). A separate safety protocol (available upon request) has been created, containing additional information on participant safety measures.

The eligibility screener can be completed in private interview rooms at study offices in downtown Toronto, using an online screener, or via telephone. For telephone-based screeners, the study staff will be instructed to keep the initial communication with potential participants as vague as possible until there is some assurance that they are in a private and safe location to hear more about the study.

Once a potential participant is deemed eligible to participate, the study staff will ask her whether she desires to continue to learn about the study and, if so, they will request her to provide informed consent to enroll. They will indicate that this process will take about 10 minutes. Informed consent can be provided immediately in person, via telephone, or via an electronic informed consent form. Staff can also arrange for a phone call at a later time to further discuss the study. The informed consent form outlines all study processes, including (1) study description, (2) a description of what is required of participants, (3) study duration, (4) expected time commitment from participants, (5) compensation, (6) data confidentiality, and (7) any potential risks or benefits of participation. Then, any questions the potential participant has will be answered by the study staff member (via telephone/in person). For electronic informed consent, contact information for the research coordinator and principal investigator will be provided in the event that participants have questions prior to providing their verbal consent or electronically signing the form. Participants will then be asked if they would like a copy of the form.

### Preintervention Assessments

As previously discussed, SCED requires the establishment of stability in a given outcome measure prior to intervention delivery [40]. We plan to assess 3 primary outcome measures that align with those assessed by previous RCT safety planning evaluations and are the direct targets of the intervention’s activities: safety self-efficacy, decisional conflict, and confidence in planning (see Table 1). These speak to the goals of safety planning, which are to *prime* those who are currently experiencing IPV to take action to improve their safety, which reflects the often nonlinear paths and ambivalence women feel about extricating themselves from a violent relationship [41,42]. Additionally, we plan to measure 2 secondary outcomes: IPV recurrence and mental health. IPV itself is a secondary outcome because changes in IPV exposure are not necessarily expected, for the reasons mentioned above. While mental health is not directly targeted by intervention activities in WITHWomen Pathways*,* participation in the intervention may secondarily lead to improvements in mental health through help-seeking actions.

**Table 1.** Outcome variables for evaluation of *WITHWomen Pathways*.

Outcome measured	Primary/secondary	Instrument	Reference
Use and perceived helpfulness of safety strategies, service use	Primary	Intimate Partner Violence Strategies Index (adapted)	Goodman et al (2003) [43]
Empowerment related to safety	Primary	MOVERS^a^	Goodman et al (2014) [13]
Decisional conflict	Primary	Decisional Conflict Scale	O’Connor (1995) [12]
Anxiety and depression symptoms	Secondary	PHQ-4^b^	Kroenke et al (2009) [44]
Intimate partner violence	Secondary	Composite Abuse Scale-Short Form	Ford-Gilboe et al (2016) [45]

a MOVERS: Measure of Victim Empowerment Related to Safety.

b PHQ-4: Patient Health Questionnaire-4.

The baseline interview is designed to last approximately 15 to 20 minutes and will end with a reminder that the participant will complete some of these same measures every 3 to 4 days for the next 2 to 3 weeks until stability is reached. Participants will receive CAD $10 (US $7.15) as an online gift card or through electronic money transfer upon finishing each preintervention interview. Following the established safety protocols for working with those experiencing IPV described above, preintervention baseline assessments can occur via telephone, in person, or via an online survey, using the mode that seems the safest for the participant [46]. For telephone-based assessments, we will also ask the participant before beginning each session if she is in a private space. Whether collected remotely or in person, the SCED data will be recorded via Zoho Survey (Zoho Corporation) and saved directly to a secure Zoho server.

After each participant’s fifth baseline interview, the research team will assess the overall score for each outcome measure. If the participant achieves stability, measured using previously cited criteria wherein at least 85% of preintervention data fall within a 15% range of the median for at least 1 primary outcome measure (Patient Health Questionnaire-4 [PHQ-4], Measure of Victim Empowerment Related to Safety [MOVERS], and Decisional Conflict Scale), they will progress to the intervention interview [47,48]. If stability is not reached after 5 assessments, baseline measurements will continue for 2 more weeks (3 additional baseline measures with CAD $10 (US $7.15) compensation at each assessment) and reassessed. If, at this point, the participant’s outcome measures are deemed stable, they will proceed to the intervention interview. If not, they will still advance to the intervention visit, but their data will not be included in the effectiveness evaluation. Using the standard SCED protocol, these assessments will result in at least 5 but no more than 8 baseline measurements, ensuring that every participant receives the intervention as soon as possible [49].

### Intervention Interview

The intervention visit is an in-person session that may last up to 90 minutes and provides detailed instructions on the WITHWomen Pathways app and its features using a study-provided tablet. During this visit, the research coordinator will inform the participant that it differs from previous visits and that an interview or survey will take place at the end of the session, featuring different questions from those asked previously. In a private, mutually agreed-upon, safe location, such as a conference room or unoccupied office either at St Michael’s hospital, at one of our partner agencies, or in the community (eg, private meeting room at a public library), the research coordinator will provide instructions on how to use the tablet and engage with the WITHWomen Pathways web app. The participant will then be allowed to explore the web app freely, with the coordinator stepping back to provide privacy. The participant will be encouraged to spend a significant amount of time (up to 30 min) exploring the web app. This includes working through all service prioritization activities and creating an individualized safety plan. The coordinator will check in periodically to answer questions. It will be explained to the participant that each time she uses the web app, it is considered a new session, and that for safety reasons, the web app does not store or restore data previously entered. After she has explored each section of the web app, the research coordinator will ensure that the participant is able to access the WITHWomen Pathways app on her own device and is comfortable using and exiting the web app from that device as well. Once the participant is comfortable and familiarized with the app, the session can be concluded. Then, the intervention visit survey will be administered using the interview guide and visual aid (available upon request). Finally, the interviewer will schedule the first remote follow-up interview and thank the participant for her time and input. Participants will be allowed and encouraged to use the web app as often as they like during and after the follow-up period.

### Postintervention Interviews

Following standard SCED protocols, the postintervention assessments use the same questionnaires and intervals as the preintervention assessments [28]. At the first postintervention assessment (scheduled during the intervention interview), the participants will complete the same outcome measures at the same intervals (every 3‐4 days) as in the preintervention assessments. Participants with children will also complete the perceived child well-being questionnaire at the final visit. All participants will complete the same number of postintervention assessments (n=8) over the course of 4 weeks after their intervention interview. Postintervention interviews can be conducted in person, via telephone, or using the online survey.

### Strategies to Prevent Attrition

Given the high participant burden of SCED and the potentially precarious position in which many women experiencing IPV find themselves, attrition from the study is likely. However, the research team has devised strategies to safely encourage participants to remain in the study. First, the study is designed to be as convenient for participants as possible, offering several modes of contact (in person, via telephone, and online), evening and weekend assessment options, and face-to-face interviews at partner organizations convenient to the participant. Compensation will also be given at the point of assessment, within a day following each check-in, and each assessment will be scheduled at the end of the preceding visit. For those who do not attend previously scheduled assessment visits, researchers will first reach out via the safe contact methods indicated by the participant at different times of the day. They will then search for updated information or attempt previously disconnected phone numbers before reaching out to the safe contacts listed by the participant. While resource limitations prevent us from overenrolling to guard against attrition, sample size is only one of several levers that SCED studies (as opposed to traditional evaluation designs) can use to improve statistical power. Should attrition become an issue, we will explore using additional baseline and follow-up measures in the remaining participants, simulating power using different effect size estimates, and assessing the number of outlier scores to understand its effect on generalizability [50].

### Managing Minor Emotional Distress

Given the topic of IPV, study population, and sensitivity of the assessment questions, researchers will undergo training on how to handle situations where participants experience emotional distress during interviews. Sufficient time will be allocated during the initial interview to address any questions or concerns, including the potential for anxiety. For telephone or in-person surveys, researchers will help participants devise a step-by-step strategy to manage various scenarios, such as how to respond if they start crying or feel uncomfortable answering a question. Participants will also discuss options for managing anxiety during the interview ahead of time, such as deep breathing or temporarily discontinuing the interview. For online links, participants will be asked to complete the survey only when they are alone, and all participants will be provided with links to mental health resources at the end of the survey. The research coordinator will be in regular contact with all participants. Researchers will also note the PHQ-4 scores of all participants and those with elevated depressive and anxiety symptoms, making sure to carefully monitor these participants specifically and provide contact information for community mental health resources. Any adverse events beyond minor emotional distress will be immediately reported to the Research Ethics Board and integrated into our assessment of the safety of using SCED for IPV interventions.

### IPV Reporting Requirements

Health care professionals and staff affiliated with St Michael’s Hospital or the University of Toronto are not legally or professionally obligated to report suspected or verified partner violence. However, reporting child abuse is mandatory under Ontario’s Child and Family Services Act, Section 125 [51]. Participants will be informed during the interview’s informed consent instructions that the interviewer is obligated to report child abuse, allowing them to control the disclosure of any such incidents.

### Data Analysis Plan

Following the SCED technical guidance published by the What Works Clearinghouse (WWC) of the US Department of Education’s Institute for Education Sciences [49], we will begin by computing descriptive statistics for each outcome measure, stratified by participant and intervention phase. For each scale, we will calculate the number of observations, mean, SD, SEM, minimum, and maximum, separately for the preintervention and postintervention periods. This will allow us to summarize overall patterns and assess changes in central tendency and variability over time.

The WWC also recommends visual inspection of data points and trend lines to gauge preintervention and postintervention effects [52]. To visualize the within-person patterns over time, we will create scatterplots for each participant and outcome measure, overlaying fitted linear trend lines for both the preintervention and postintervention phases. This will allow for visual inspection of potential shifts in level and trend following the intervention. A vertical reference line will denote the point of intervention onset.

Internal consistency will be assessed using Cronbach α, calculated on the basis of the preintervention responses for each scale described previously. These estimates will provide an indication of scale reliability prior to any intervention effects.

To quantify the pre-post changes in outcomes for each participant, we will follow the WWC guidance by calculating Tau-U, a nonparametric effect size metric. Tau-U allows for the estimation of intervention effects while accounting for trends that may be present during the baseline (preintervention) phase [53,54]. For each outcome and participant, we will compute (1) Tau (preintervention, A), to evaluate baseline trend; (2) unadjusted Tau-U (A vs postintervention, B), which compares data points between the baseline and postintervention phases without adjusting for baseline trend; and (3) adjusted Tau-U (A vs B), which corrects for any nonrandom trends in the baseline phase. Consistent with Fingerhut et al [54], our primary aim is not to assess statistical significance, which would be unreliable in small sample size designs such as SCED, but instead to interpret the direction and magnitude of observed effects. We will use previously validated benchmarks to classify effect sizes as small (0.20‐0.59), moderate (0.60‐0.79), or large (≥0.80) [53]. Even small effect sizes will be considered potentially meaningful when supported by consistent patterns observed through visual inspection of the data (see Table 2).

**Table 2.** Five visual analysis measures of comparison typically used for single-case experimental design evaluation.

Measure	Definition
Nonoverlap of all pairs (NAP)	Values from baseline and intervention time points are paired, and a percentage of pairs whose values do not overlap is calculated.
Extended celeration line (ECL)	An NAP that controls for positive trends in the preintervention phase by calculating the proportion of postintervention data points that are above the median trend line plotted from preintervention data.
Improvement rate difference	It is calculated as the difference in the rates of increase in outcome variable data between the preintervention and postintervention phases by dividing the number of “improved data points” by the total number of data points in that phase.
Tau-U	Tau-U adjusted pairs each preintervention and postintervention observation to make n pairs. The number of positive-change, negative-change, and tied pairs is calculated for each outcome variable
Percentage of nonoverlapping data, corrected (PNDC)	Particularly useful when anticipating an increase in the outcome variable postintervention, PNDC locates the highest data point in the preintervention phase and calculates the percentage of postintervention data points that exceed it. A data correction procedure is used to control for an upward preintervention trend.

### Handling Missing Data

SCED requires multiple data collection points with each participant, increasing the likelihood that one of these data collection opportunities will be missed. In the event of interim missing assessments (ie, a missed data point from an enrolled participant), we will still attempt to establish stability. To do so, we will leave a break in the time-series line for visual analysis; data points will not be connected across a missing observation. For the nonparametric effect size calculation (Tau-U), comparisons will be made using all available preintervention and postintervention data pairs, as the statistic does not require an uninterrupted series.

### Data Variability

To address potential variability in the data, our visual analysis will systematically examine the stability of data points within each phase, in addition to the level and trend. This will be supplemented by our calculation of SD for each outcome per participant in both preintervention and postintervention periods. This allows us to assess not only the occurrence of a change but also the consistency of the intervention’s effect, even with variable data in the preintervention or postintervention periods.

## Results

Recruitment commenced on July 3, 2024. As of December 2025, a total of 4 participants have completed the study. Data analysis has been completed for the 4 participants. Additional recruitment is underway, and results are expected to be published in spring 2026.

## Discussion

The aim of this study is to assess the viability of an innovative evaluation approach for a localized safety planning web app among English-speaking women currently (or until very recently) experiencing IPV in the GTA. To the best of our knowledge, this study will be the first to evaluate an IPV resource using SCED, and the small sample size will allow the research team to ethically monitor women in precarious situations while still collecting robust data.

### Other Alternatives to RCTs

SCED is not, however, the only alternative to RCTs for gauging the effectiveness of app-based safety planning interventions. We also considered whether stepped wedge designs [55] and interrupted time series (ITS) designs could feasibly be used to assess WITHWomen Pathways. We decided against a stepped wedge study given that it requires more participants and longer preintervention baseline phases for some participants versus others. Because our second aim is to understand how to more quickly establish baseline stability to provide the intervention as quickly as possible, we believe that it would be unethical to withhold the intervention from some participants given our inclusion criteria. Similarly, ITS designs either require the use of a control group with which the intervention is compared (controlled ITS) or the use of a counterfactual, which assumes that the outcome measure in the group exposed to the intervention would have remained the same in absence of the intervention [56]. We consider the use of a control group for those experiencing IPV as potentially ethically dubious and therefore did not consider a controlled ITS. Furthermore, ITS studies are best suited to understand population-level effects, which—while making them potentially more generalizable—reduces the ability to understand how an intervention impacts the safety planning behaviors of an individual and the IPV experienced by them. For hyperlocal interventions such as WITHWomen Pathways, we believed that a more individualized approach would provide more helpful information on how local community members might be aided by the intervention. Therefore, while stepped wedge and ITS studies may be helpful in future larger trials, understanding the feasibility of SCED is an important step toward improving the rigor of IPV intervention evaluation.

### Study Limitations

There are some notable limitations to this study. First, research activities require that women be able to speak and read English, which excludes non-English speakers and women with visual impairment. Second, with its multiple points of intervention and smaller sample size, SCED has safety and statistical limitations. First, having multiple contact points with each participant increases the chance that the abusive partner could become aware of her participation, increasing the risk of IPV. To address this, we have developed strict safety protocols and can promptly leverage our connections with local service providers should emergencies arise. SCED approaches also have multiple measurements in close temporal proximity, increasing participant burden. To help limit attrition, we will clearly communicate with potential participants about the time involved at the outset; invest in training for study team members to promote friendly, empathetic connection with participants; and provide fair compensation for the demands on participants’ time. Statistically, SCED carries four major risks: (1) the potential for a lack of variability in the preintervention and postintervention measures because measurements are completed at short intervals, (2) limited generalizability due to a small number of cases, (3) threats to internal validity due to test bias and social desirability bias [40], and (4) potential confounding effects due to the lack of randomization. To address risk 1, we will use the WWC recommendations for conducting sensitivity analyses and reporting multiple effect size estimators (eg, multiple visual analysis methods). Regarding risk 2, although multiple-case SCED (when rigorously evaluated) can be generalizable in many of the same ways as RCTs, this necessitates replication, which requires additional resources [57]. We do not anticipate this preliminary trial to be generalizable. Rather, it is intended to better identify the potential utility of SCED protocols in the evaluation of IPV-related interventions and adaptations that may be necessary. Regarding risk 3, we will use the percentage of nonoverlapping data, corrected (PNDC), to ascertain patterns of preintervention increase, as recommended by the WWC (see Table 2). Regarding risk 4, once this preliminary trial is complete, we intend to introduce randomization to different permutations of the WITHWomen Pathways app in future studies.

### Research Plans

Should SCED show the promise for rigorous evaluation of IPV interventions that we anticipate, we intend to conduct a rigorous, multiple-baseline SCED to measure the impact of WITHWomen Pathways on the primary outcomes of interest in this study before integrating the app into existing collaborations with GTA IPV service organizations. Our network of more than 2 dozen such service providers in the area allows for the rapid scale-up of WITHWomen Pathways for overburdened agencies who currently have no evidence-based safety planning tools to recommend to women who feel unsafe, but who are unwilling or unready to leave their relationship. Alongside the scale-up of the English version, we plan additional SCED evaluations of the Spanish and French versions of WITHWomen Pathways, as are adaptations for recent immigrants and asylum seekers who may also be reticent to engage with formal services [58]. This stepped approach allows us to move forward with implementation while creating stand-alone, tailored versions of the tool for women in the GTA who cannot easily avail of the current version of WITHWomen Pathways. Additionally, we anticipate that this study will facilitate a more widespread use of SCED in IPV intervention evaluation.

### Conclusions

The findings of this study could have important implications for women and domestic violence service organizations in the region. Improved safety planning is a key marker of future help-seeking for IPV and may help extricate women in violent relationships prior to the escalation of violence to more serious and life-threatening actions [24]. Using WITHWomen Pathways in conjunction with other domestic violence services could also improve service delivery for organizations whose resources and staff are thinly stretched, allowing these organizations to assist more women by providing safety planning resources to women who cannot or choose not to engage with more formal services (eg, law enforcement). By testing the viability of using SCED to assess the effectiveness of WITHWomen Pathways, we may be able to assist women currently navigating violent relationships, by providing rigorously tested safety planning information that is safe to use, tailored to the local context, and self-directed.

## Abbreviations

COPE: Committee on Publication Ethics
GTA: Greater Toronto Area
IPV: intimate partner violence
ITS: interrupted time series
MOVERS: Measure of Victim Empowerment Related to Safety
PHQ-4: Patient Health Questionnaire-4
PNDC: percentage of nonoverlapping data, corrected
RCT: randomized controlled trial
REB: Research Ethics Board
SCED: single-case experimental design
WWC: What Works Clearinghouse
